# Invasive bacterial co-infection in African children with Plasmodium falciparum malaria: a systematic review

**DOI:** 10.1186/1741-7015-12-31

**Published:** 2014-02-19

**Authors:** James Church, Kathryn Maitland

**Affiliations:** 1 Department of Paediatrics, Faculty of Medicine, Imperial College, Wellcome Trust Centre for Clinical Tropical Medicine, St Mary’s Campus Norfolk Place, London W2 1PG, UK; 2 KEMRI-Wellcome Trust Research Programme, Kilifi, Kenya; 3 Department of Paediatrics, Queen Mary, University of London, Mile End Road, London E1 4NS, UK

**Keywords:** Severe malaria, Invasive bacterial infection, Gram-negative organisms, Children, Mortality, Epidemiology, Africa

## Abstract

**Background:**

Severe malaria remains a major cause of pediatric hospital admission across Africa. Invasive bacterial infection (IBI) is a recognized complication of *Plasmodium falciparum* malaria, resulting in a substantially worse outcome. Whether a biological relationship exists between malaria infection and IBI susceptibility remains unclear. We, therefore, examined the extent, nature and evidence of this association.

**Methods:**

We conducted a systematic search in August 2012 of three major scientific databases, PubMed, Embase and Africa Wide Information, for articles describing bacterial infection among children with *P. falciparum* malaria using the search string ‘(malaria OR plasmodium) AND (bacteria OR bacterial OR bacteremia OR bacteraemia OR sepsis OR septicaemia OR septicemia).’ Eligiblity criteria also included studies of children hospitalized with malaria or outpatient attendances in sub-Saharan Africa.

**Results:**

A total of 25 studies across 11 African countries fulfilled our criteria. They comprised twenty cohort analyses, two randomized controlled trials and three prospective epidemiological studies. In the meta-analysis of 7,208 children with severe malaria the mean prevalence of IBI was 6.4% (95% confidence interval (CI) 5.81 to 6.98%). In a further meta-analysis of 20,889 children hospitalised with all-severity malaria and 27,641 children with non-malarial febrile illness the mean prevalence of IBI was 5.58 (95% CI 5.5 to 5.66%) in children with malaria and 7.77% (95% CI 7.72 to 7.83%) in non-malaria illness. Ten studies reported mortality stratified by IBI. Case fatality was higher at 81 of 336, 24.1% (95% CI 18.9 to 29.4) in children with malaria/IBI co-infection compared to 585 of 5,760, 10.2% (95% CI 9.3 to 10.98) with malaria alone. Enteric gram-negative organisms were over-represented in malaria cases, non-typhoidal Salmonellae being the most commonest isolate. There was weak evidence indicating IBI was more common in the severe anemia manifestation of severe malaria.

**Conclusions:**

The accumulated evidence suggests that children with recent or acute malaria are at risk of bacterial infection, which results in an increased risk of mortality. Characterising the exact nature of this association is challenging due to the paucity of appropriate severity-matched controls and the heterogeneous data. Further research to define those at greatest risk is necessary to target antimicrobial treatment.

## Background

Malaria remains a leading cause of childhood morbidity and mortality worldwide, accounting for 7% of deaths in children younger than five years of age [1]. In the past few years two of the largest clinical trials ever conducted in African children with severe malaria (SM) have concluded, both holding major implications for treatment guidelines [2,3]. With the introduction of artesunate in sub-Saharan Africa (sSA), baseline mortality in children with SM will at best be between 6% to 8.5% but likely to be substantially higher outside the framework of Good Clinical Practice-run trials. Understanding the key correlates of poor outcome may identify future targets for additional definitive or adjunctive treatments.

Over time there has been a piecemeal accumulation of data indicating that children with *Plasmodium falciparum* malaria are at risk of invasive bacterial infection (IBI). Blood stream infection, largely secondary to enteric Gram-negative organisms (EGNOs), with a predominance of non-typhoidal salmonella (NTS) species, has been widely reported as a complication of severe malaria. However, it remains uncertain whether malaria infection is a risk factor for invasive bacterial disease since the majority of children in malaria-endemic Africa are frequently infected by *P. falciparum* throughout childhood and only a minority will develop severe disease. A sub-analysis within a comprehensive systematic review of blood stream infections in Africa indicated that 6.5% of 11,814 malaria infections had concomitant bacteremia [4]. Which children are at greatest risk of developing dual infection and whether this extends across the clinical spectrum (asymptomatic, mild and severe) remains unclear.

This is important for two reasons. Firstly, concomitant IBI in children hospitalized with evidence of recent or intercurrent malaria, with or without severe manifestations, results in a significantly worse outcome. In one epidemiological study an estimated one third of all SM deaths were attributable to bacteremia [5]. Secondly, it has implications when considering revisions of current management guidelines to include routine provision of antibiotics to all children with malaria. Routine antibiotics, along with anti-malarials, are currently recommended for children with SM [6]. However, malaria remains a very common cause of pediatric admission and the indiscriminate use of antibiotics would be both financially costly and could perpetuate the rise of antibiotic resistance. Thus, there remains a need for clarity on whether children with malaria are more susceptible to a bacterial infection and its precise nature in order to tailor antimicrobial management. In this systematic review we bring together the current breadth of published data on the prevalence of IBI among African children with malaria.

We reviewed published observational and epidemiological data in order to establish whether there was evidence of an association between malaria and IBI. We sought to determine the prevalence of co-infection in children with malaria infection, the risk factors for co-infection and the mortality effect in African children, drawing on this knowledge to consider the implications for future treatment guidance.

## Methods

Studies were eligible for inclusion in the review if they were conducted among children with malaria, who had blood cultures taken and were admitted to hospitals or outpatient clinics in sSA. Although we restricted our review to studies reporting bacteremia, we did not exclude studies describing other bacterial co-infection, such as urinary tract infection or meningitis.

We performed a systematic search for articles describing bacterial infection among children with *P. falciparum* malaria. Search terms used were ‘(malaria OR plasmodium)’ AND ‘(bacteria OR bacterial OR bacteremia OR bacteraemia OR sepsis OR septicaemia OR septicemia)’ in three major scientific databases, PubMed, Embase and Africa Wide Information (AWI). The Internet search was carried out on *13 and 14 August 2012*. Searches of PubMed and Embase were limited to humans while AWI was limited to humans and scholarly (peer reviewed) journals. Abstracts and titles from all years were compiled in Endnote (Thomson Reuters) and screened, after the removal of duplicates. Prospective studies were required to recruit children systematically or consecutively and to evaluate all cases. Our review was restricted to articles either written in English or translated into English. Unpublished data were not included. The protocol and review are not registered.

Eligible publications were retrieved in full text if available online or from academic libraries. Both prospective and retrospective case series of children with malaria undergoing blood culture were subdivided according to principal inclusion criteria (SM, all malaria or non-severe malaria). Studies principally reporting epidemiological associations were tabled separately. The definitions of SM varied widely. In order to facilitate agreement between the reviewers for study inclusion we used the following criteria for assessment of the quality of studies reporting on severe malaria (SM): 1) criteria for SM well-defined; 2) bacterial species defined or quality assurance verified that contaminants were excluded; 3) mortality data for ‘high risk’ SM cohort at least 7.4% (the lowest 95% confidence for artesunate arm of the AQUAMAT multicountry trial [2]; the overall mortality in this arm was 8.5% (7.4% to 9.5%)) [2]. These were ranked as low, moderate and high (1, 2 and 3) according to the number of criteria met. We reviewed the reference lists from articles rated ‘high quality’ to identify additional secondary articles not found during the initial online search.

Both authors examined the manuscripts and excluded studies during abstract review if they did not include a cohort of patients with a confirmed diagnosis of malaria, case reports, review articles or behavioral studies. During full text review, we excluded studies if they were conducted outside of sSA, did not report any microbiological data or featured predominantly adults. Studies reporting a mixed adult child population were only included if the data for children could be readily separated. Studies in which relevant data could not be extracted or where inclusion criteria failed to represent a population of children with malaria, were also excluded. For study sites generating several papers from the same study cohort, we included only the most appropriate study unless time periods varied. Data on neonates were excluded since neonates represent a group largely unaffected by malaria but with an unrelated, distinct vulnerability for IBI. We did not actively consult with specialists in the field to discuss or review.

For each study we worked out case fractions for malaria IBI co-infection as ‘number of children with malaria AND IBI’ (numerator) divided by ‘number of children with malaria’ (denominator). We used the Q test, as a measure of heterogeneity among studies, calculated as the weighted sum of squared differences between individual study effects and the pooled effect across studies. I^2^ was calculated to quantify heterogeneity (between-studies variability) [7]. Since the definitions for SM vary from study to study, we also compared the study inclusion criteria for SM cases with the WHO definition of SM [8].

## Results

**Figure 1:**
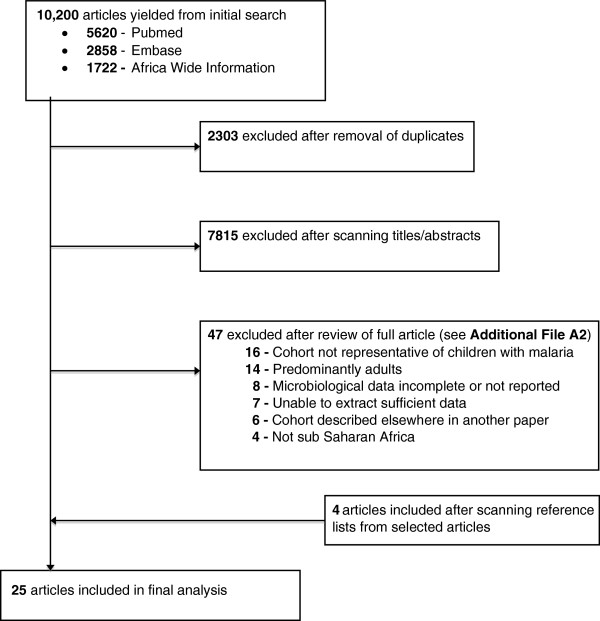
**PRISMA flow diagram.** Legend: nts, non-typhoidal salmonellae; str.pn, *Streptococcus Pneumoniae;* h. inf, *Haemophilus Influenzae;* s. typh, *Salmonellae Typhi;* gm neg, Gram negative organisms; gm pos, Gram positive organisms.

Our search of the published literature yielded 10,200 articles and 7,897 unique articles following removal of duplicates. After reviewing the abstracts 72 full text reports were identified for closer evaluation, which were reduced to 21 studies after applying the exclusion criteria (Figure 1).

We excluded 15 studies since they described specific populations not primarily defined by malaria status. Studies alluding to an association between malaria and IBI but with incomplete information or unsuitable study design were tabulated separately (Additional file 1: Table S2). Only four studies were excluded due to location outside of sSSA, highlighting the dearth of data from other regions. Three of these studies reported predominantly data from adults [9-11]. A study of 340 children with severe malaria in Papua New Guinea recorded two cases of malaria with concomitant infection (invasive candida and *Klebsiella Pneumoniae*); however, not all children had blood cultures taken [12]. Other reasons for exclusion are detailed in Additional file 1: Table S1.

A further four papers were found from scanning reference lists of selected articles, leaving 25 studies from a total of 11 sSA countries. They comprised twenty cohort analyses (including inpatient and outpatients studies), two randomized controlled trials and three epidemiological studies, summarized in Tables 1 and 2 according to study type and clinical severity.

**Table 1:** **Summary of 22 observational studies recording invasive bacterial infection (IBI) in children with *Plasmodium falciparum *malaria in sSA**

**Author**	**Location**	**Study period**	**Study type; primary inclusion criteria. Hospital type**	**Age range**	**Number in study**	**Cases with malaria Number**	**Cases with IBI *number***	**Case fraction with malaria IBI co infection**	**Predominant organisms IBI Co-infection**	**Comments or associations**
								**n/N (%)**		
**SEVERE MALARIA**										
Angyo [13]	Jos, Nigeria	Aug 92 – Oct 93	PCS;	1 m-14y	501	501	35	7.0%	SPN 34.3%	SM definition included slide negative cases (n = 214). SM mortality = 3.2%.
			Severe malaria. Urban TH						*S. aureus* 20%	
									Salm spp 14.3%	
Bassat [23]	Manhica, Mozambique	Jun 03 – May 07	RCS;	0-5y^a^	1,780	1,404 with BC data	76	5.4%	All EGN 36.8% NTS 15.8%	High mortality SMA and IBI (31%).
			Severe malaria. Rural DH						SPN 26.3%.	Valid BC data excludes contaminants
									*S. aureus* 17%,	
Berkley [36]	Kilifi, Kenya	Apr 93 – May 96	RCS	NR	783	643 with BC data	42	6.5%	SPN 30%	Bacteremia less prevalent in >30 m (2.5%).
									*S. aureus* 16%	
			Severe malaria. Rural DH						NTS 14%	
Berkley [24]	Kilifi, Kenya	Aug 98 – Jul 02	PCS	>60d	3,068	2,048	127	6.2%	EGN at high parasite counts >50,000	Mortality strongly correlated with IBI (but not with SPN and HIb).
			Severe malaria. Rural DH							Case fatality inversely related to parasitemia.
Bronzan [38]	Blantyre, Malawi	1996 - 2005	PCS;	>6 m	1,388	1,388	64	4.6%	NTS 58%	Invasive NTS assoc with severe anemia (73% of NTS cases). Not associated with mortality.
		(Rainy seasons)	Severe malaria. Urban RH						SPN 11%	
Dorndorp [2]	**Multisite**	Oct 05 –	RCT;	1.6-4.3y	5,425 Slide +/or	657 with BC data	65	9.9%	NR	60% children received antibiotics.
	Mozambique	Jul 10	Severe malaria.		RDT + ve					
	Gambia		Multi center							
	Kenya DRC									
	Ghana									
	Tanzania									
	Uganda									
	Nigeria									
	Rwanda									
Enwere [39]	Banjul, Gambia	1992-1994	RCT; Cerebral malaria	1-9y	576	276 with BC data	14	5.1%	*S. aureus* 42.9%	Possible contaminants. High survival rate without antibiotic treatment.
			(BCS ≤2) Urban TH						EGN 28.6%	
Evans [22]	Kumasi, Ghana	nr	PCS;	4 m-11y	251	182 with BC data	23	12.6%	NTS 43%	OR for IBI 3.5 (95% CI 1.4-8.2). No positive association of SM and bacteremia.
		Pub 2004	Severe malaria. Urban TH						S*.aureus* 39%	
									*E.coli* 4%	
Kremsner [42]	Lambarene, Gabon	nr	PCS; sub-analysis of Abx trial; Severe malaria	>6 m	100	59 with	7	11.9%	*S. aureus* (3) 42.9%	Addition of clindamycin to quinine shortened disease course
		Pub 1995	Rural DH			BC data			EGN (2) 28.5%	
Prada [43]	Lagos, Nigeria	May 92 – Aug 92	PCS; Cerebral malaria	4 m-6y	50	50	8	16%	All EGN 75%	Hemolytic activity detected in 50% of bacterial strains analyzed
			Urban TH						Salmonella 25%	
**ALL SEVERITY MALARIA**										
Akinyemi [44]	Lagos, Nigeria	Oct 04 – Sept 05	PCS; Febrile illness.	0-15y^a^	235	60	5	5/60	*S. tyhpi* 45.2%	Only salmonella isolates reported. Included adults Child results reported here (unless indicated).
									*S. enteritidis* 24.2%	
			2 urban RHs & 2 urban clinics					(8.3%)	(Incl adults)	
Akpede [40]	Benin, Nigeria	Oct 88 – Oct 89	PCS;	1 m-5y	642	446	67	43/446	*S. aureus* 39.5%	Reanalysis of data. Retracted original suggestion of association of IBI and malaria. Note case fraction of IBI in malaria inaccurate (non-malarial febrile illness not differentiated)
			Signs of febrile illness					(9.6%)	EGN 23.2%	
			Urban TH							
Bahwere [25]	Lwiro, DRC	Jan 89 – Dec 90	RCS;	NR^a^	779	182	124	45/182	All patients^b^	Positive malaria film and anemia (Hb < 8) associated with higher IBI case fraction
			All hospital admissions. Rural DH					(24.7%)	NTS 36.5%,	
									*E. coli* 15.1%, Citrobacter 6.3%	
Berkley [52]	Kilifi, Kenya	Feb 99 – Dec 01	RCS;	>60d	11,847	5,270	843	157/5,270 (3.0%)	NR	Source data (Berkley 2009) [24]
			All hospital admissions. Rural DH							1/9 with syndrome indicating Abx Rx had IBI.
Mabey [26]	Fajara, Gambia	Jan 79 – Oct 84	RCS;	NR	5,466	426	146	43/426	NTS 69.8%	Seasonal association of iNTS with malaria. NTS rare (3%) in kids >4y. iNTS more common in malaria season.
			All hospital admissions. Rural DH			incl recent malaria		(10.1%)	S. typhi 11.6%	
Mtove [45]	Muheza, Tanzania	Mar 08 – Feb 09	PCS;	2 m-14y	1,502	947	156	75/947	All patients^b^	Invasive NTS more likely to have malaria, recent malaria, anemia, low glucose
			Febrile illness + ≥1 severity criteria.			incl RDT + ve		(7.9%)	NTS 29%	
									*E. coli* 17%	
			Rural DH						*S. typhi* 9%	
Nadjm [5]	Muheza, Tanzania	June 06 - May 07	PCS;	2 m-13y	3,639	2195	341	100/2,195 (4.6%)	NTS 52%	>50% organisms not susceptible to 1st line Abx. Case fatality non-malaria IBI = 19%
			Fever in last 48 h.							
			Rural DH							
Okwara [20]	Nairobi, Kenya	Jan 01 – Mar 01	CSS;	3 m-12y	264	158	32	18/158	Any GNR 62.5%	Also examined urinary isolates. 60% malaria dx in non-endemic area
			Febrile Illness.					(11.4%)	NTS 34.4%	
			Urban TH							
Sigauque [71]	Manhica, Mozambique	May 01- April 06	RCS;	0-15y^a^	23,686	10,699	1,550	621/10,699 (5.8%)	All patients^b^	Almost half of the community acquired bacteremias had concomitant malaria
			All hospital admissions. Rural DH						NTS 26%	
									SPN 25%	
									*S. aureus* 12%	
									*E. coli* 10%	
Were [41]	Siaya, Kenya	Mar 04 – Jan 06	PCS; Malaria admissions;	1 m-36 m	585	585	59	59/506	NTS 42.3%	Proportion of IBI decreased with increasing parasitemia. Excluded previous hospitalization and CM.
								(11.7%)	*S. typhi* 37%;	
			Rural DH						*S. aureus* 35.6%	
**NON-SEVERE MALARIA**										
Ayoola [27]	Ibadan, Nigeria	Jun – Nov 98	PCS;	1 m-12 m	102	47	39	16/47	Gram pos 56%	Excluded SM and if history of antibiotic use in preceding 7 days
			Fever. Urban TH					(34%)	Gram neg44%	
Brent [28]	Kilifi, Kenya	May 03 – Oct 03	PCS; Unselected outpatient attendees.	0-5y^a^	1093	480	22	7/480	All patients^b^	Recruitment outside of malaria season. Excluded children with recent admission (10 days)
			Rural DH					(1.5%)	SPN 50%	

^a^Study population may have included neonates - unable to extract information from the text; ^b^microbial prevalences refer to all study patients (not only those with malaria and IBI). *Abx,* antibiotic; *BC,* blood culture; *CAB,* community acquired bacteremia; *CCS,* cross sectional survey; *DH,* district hospital; *MH,* mission hospital; NR*,* not reported; *PCS,* prospective case series; *RCS,* retrospective case series; *RCT,* randomized controlled trial; RDT, rapid (malaria) diagnostic test; RH*,* referral hospital; *Rx,* treatment; TH*,* teaching hospital. Organisms: *EGN,* enteric gram negatives; *GN,* gram negative organisms; *GPO,* gram positive organisms; *HIb, Haemophilus influenzae* Group B; NTS*,* non-typhoidal salmonellae; Salm spp, salmonellae species; SPN*, Streptococcus pneumoniae*. Malaria definitions: IC*,* impaired consciousness; RD*,* respiratory distress; SMA*,* severe malaria anemia.

**Table 2:** Epidemiological studies reporting the incidence of malaria and IBI infection over time

**EPIDEMIOLOGICAL STUDIES**									
**Author**	**Location**	**Study period**	**Study type; primary inclusion criteria**	**Age**	**Change in incidence of malaria BSI**	**Change in incidence of IBI *Per 100,000 person years***	**Case-fraction of IBI co-infection**	**Predominant organisms for IBI co-infection**	**Comments**
Mackenzie [32]	Fajara and Basse, Gambia	1979 – 2008 Multiple time points	Retrospective comparative case series; 4 data sets: BC taken from unwell children or suspected IBI. Routine malaria slides for febrile children	Most <5y	*% Malaria in febrile children*	Fajara: 60 to 10 (1979 to 2005)	NR	NR	NTS declined in parallel with malaria but pneumococcal bacteremia did not.
					Fajara: 33% to 6% (1999 to 2007)	Basse: 105 to 29 (1989 to 2008)			
					Basse: 45% to 10% (1992 to 2008)				
Mtove [33]	Muheza, Tanzania	2006 – 2010	Cumulative data from three prospective case series; Severely ill febrile children	2 m-14y	547 to 106/*100,000 person years*	Overall from	167/1,898 (8.8%)	*All patients*^*a*^	Severe malaria only. S. typhi most common in older age group (5-14y), and increased with decreasing malaria.
						184 to 60:		NTS 34%	
						NTS: 82 to 7			
						SPN: 34 to 7			
						HIb: 21 to 4			
						S. typhi: 7 to 15			
Scott [31]	Kilifi, Kenya	1999 - 2009	Case control and longitudinal study; Cases: hospital admissions with bacteremia. Controls: children born in study area.	0-13y	28.5 to 3.45*/1,000 person years*	2.59 to 1.45*/1000 person years*	NR	*All patients in matched case–control study*^*a*^	Reduction in protection afforded by HbAS in parallel (*P = 0.0008*). 62% bacteremia cases attributable to malaria
								All GNs 33.2%	
								SPN 38%	
								NTS 20.9%	

^a^microbial spectra refer to *all study patients* (not only those with malaria and IBI) ***}*** *Abx*, antibiotic; BC, blood culture; CAB, community acquired bacteremia; *CCS*, cross sectional survey; *DH*, district hospital; IBI, invasive bacterial infections; HbAS, Sickle cell trait *MH,* mission hospital; *NR*, not reported; *PCS*, prospective case series; *RCS*, retrospective case series; *RCT,* randomized controlled trial; RDT, rapid (malaria) diagnostic test; *RH,* referral hospital; *Rx,* treatment; *TH*, teaching hospital. Organisms: *EGN*, enteric gram negatives; *GN*, gram negative organisms; *GPO,* gram positive organisms; *HIb, Haemophilus influenzae* Group B; *NTS*, non-typhoidal salmonellae; *Salm* spp, Salmonellae species; *SPN, Streptococcus pneumoniae.*

### Evidence of IBD co-infection in malaria

Table 1 summarizes the data from 22 studies reporting the incidence of bacteremia in children with malaria in sSA, subdivided according to malaria case definition (severe malaria, all-severity malaria and non-severe). Information on location, study period, hospital type, case fraction of malaria-IBI, predominant organisms causing IBI and mortality data is included. In total, 21 studies described hospitalized inpatients and 14 were conducted prospectively.

### Severe malaria

The first section of Table 1 summarizes the details of the 10 studies reporting data on children admitted with severe malaria over the period 1992 to 2010 from 15 centers in 11 sSA countries. In total, these involved 7,208 children, including 461 with concomitant IBI, mean case fraction 6.4% (95% confidence interval (CI) 5.81 to 6.98%). Eight studies involved children with all types of severe malaria, including one randomized controlled trial, whereas two only included children with cerebral malaria. With one exception [13], all studies used a positive blood slide as part of their severe malaria definition. The data from these 10 studies are summarized in Table 3, together with an assessment of the study quality. The case fraction with IBI varied according to study quality from 6.01% (95% CI 5.27 to 6.74) in the highest ranked studies including 256 bacteremias in 4,261 cases to 8.2% (95% CI 5.92 to 24.26%) involving 50 bacteremias in 610 children in the lowest ranked studies (Table 3). The Q test and I^2^ statistic were 40.1 and 77.5, respectively, indicating substantial heterogeneity and between-study variability; thus, a formal meta-analysis was not performed. Of note, other studies, not included in this table, described other bacterial infections (pneumonia [14,15], meningitis [16-18] and urinary tract infections [19-21]) complicating malaria. Only three studies included information on a non-malaria comparator group [22-24].

**Table 3:** Quality assessment of studies describing children with severe malaria (SM) and concomitant invasive bacterial infection (IBI) with calculated case fractions and case fatality rates

**Study**	**Study inclusion criteria for severe malaria compared to WHO definition**^**a**^	**N with IBI data**	**% without IBI data**	**Overall mortality rate**	**n IBI**	**Case fraction (%) with IBI**	**Case fatality malaria with concomitant IBI (%)**	**Case fatality malaria without IBI (%)**	**Overall rating of study quality**	**Ranking**
				**severe malaria (%)**		**co-infection**				
						**(95% CI)**				
Berkley [16]	IC or RD + parasitemia (one slide) Excludes SMA without IC or RD	643	18%	89/783 (11.4%)	42	**6.53 (4.56-8.51)**	14/42 (33%)	52/498 (10%)	High	YYY
Berkley [24]	IC or RD or SMA + parasitemia (any of three slides)	2,048	0.5%	76/861 (8.8%)	127	**6.2 (5.12-7.73)**	36/127 (28%)	137/1921 (7.1%)	High	YYY
Bronzan [38]	WHO plus asexual parasitemia (slide repeated six hrly). Clinical signs do not include RD	1,388	5.3%	235/1,388 (16.9%)	64	**4.61 (3.48-5.74)**	14/64 (22%)	211/1324 (16%)	High	YYY
Evans [24]	WHO definition plus asexual parasitemia (one slide)	182	0%	16/182 (8.8%)	23	**12.64 (7.47-17.8)**	2/23 (8.7%)	14/159 (8.8%)	High	YYY
**Subtotal**		**4,261**		**416/3,224 (12.9)**	**256**	**6.01 (5.27-6.74)**				
Bassat [23]	WHO signs (incl glucose <2.2 mmol/L) + asexual parasitemia (one thick film)	1,404	12%	81/1,648 (4.9%)	76	**5.41 (4.2-6.63)**	13/59 (22%)	50/1244 (4.0%)	Moderate	YYN
Dondorp [2]	WHO definition plus RDT positive; requiring parenteral treatment	657	NR (88%) (BC data not available in all patients)	527/5,425 (9.7%)	65	**9.89 (7.49-12.3)**	NR	NR	Moderate	YNY
Enwere [39]	Cerebral malaria *(BCS < =2)* plus asexual parasitemia)	276	52% (BCx data not consecutive)	122/576 (21.1%)	14	**5.07 (2.42-7.73)**	1/14 (7.1%)	121/562 (22%)	Moderate	YNY
***Subtotal***		**2,337**			**155**	**6.63 (5.59-7.68)**				
Angyo [39]	Clinical signs also include hypoglycemia; shock; DIC; acidosis; hemoglobinuria. Did not include confirmed parasitemia.	501	NR	16/501 (3.2%)	35	**6.99 (4.67-9.30)**	NR	NR	Low	YNN
Kremsner [42]	Clinical signs also include hyperparasitemia, glucose <2.2. Excludes children with Sickle Cell disease.	59	41% (BCx data not consecutive)	2/100 (2%)	7	**11.86 (3.08-35.1)**	1/7 (14%)	0/52 (0%)	Low	YNN
Prada [43]	Cerebral malaria only (not defined)	50		NR	8	**16.0 (4.91-27.09)**	NR	NR	Low	NYN
***Subtotal***		**610**			**50**	**8.2 (5.92-24.26)**				
**Overall total**		**7,208**			**461**	**6.40 (5.81-6.98)**	**81/336**	**585/5760**		
							**(24.1%)**	**(10.2%)**		

^a^WHO Definition = clinical symptoms suggestive of SM according to WHO guidelines which includes children with the following symptoms or signs: 1) a Blantyre coma score (BCS) of two or less; 2) severe anemia with a hemoglobin level <5 g/dl or a hematocrit <15%; 3) severe respiratory distress, being marked in-drawing of the lower chest wall or deep (acidotic) breathing; and 4) prostration, being the inability to sit upright in a child able to do so, or to drink in the case of children too young to sit. Ranking is based on: 1) *c*riteria for SM clearly defined*;* 2*) b*acterial species defined and contaminants excluded*; and* 3*) m*ortality rate >7.4*%* (lowest CI in AQUAMAT) for SM cases*.* High = YYY, Moderate = YYN/YNY/NYY; Low = NNY/NYN/YNN for each of the criteria. BC, blood culture; *NR*, not reported; RDT, rapid (malaria) diagnostic test; Malaria definitions; IC*,* impaired consciousness; RD, respiratory distress; SMA, severe malaria anemia.

### All severity malaria: hospital studies

The second section of Table 1 summarizes data from another 10 studies that were largely prospective studies of febrile children admitted to health facilities. In total, these 10 studies included 20,889 children with malaria and 27,641 children with non-malarial febrile illness as a comparator group (eight studies only). Bacterial infection was present in 1,166 children with malaria, 5.58% (95% CI 5.50 to 5.66), and 2,148 children without malaria, mean case fraction 7.77%, (95% CI 7.72 to 7.83%). In six out of eight studies with comparator groups, bacteremia was more common in the non-malarial febrile children (Table 4). Based on the eight studies concurrently reporting prevalence of IBI in 20,323 children hospitalized with malaria (n = 1,102; 5.4%; (95% CI 5.3 to 5.5) and 27,641 children with non-malarial febrile illness (n = 2,148; 7.77%) (Table 4), we derived a malaria attribution case fraction (MAF), indicating that children with malaria were less likely to have IBI than children with other causes of febrile illness (MAF = -2.35%). Two studies reported a higher case faction of malaria co-infection than non-malarial febrile illness in the Democratic Republic of the Congo (DRC) [25] (24.7% versus 13.0%) and in the Gambia (10.1% versus 1.97%) [26]. The latter study also included recent cases of malaria (defined as the presence of *P. falciparum* gametocytes and/or pigment on a blood film). However, interpreting data from these studies, which used hospitalized children with non-malarial febrile illness as a comparator group, one must consider the possibility of selection bias. If the threshold for admission in children with confirmed malaria is lower, whereas children with a non-malarial febrile illness require one or more manifestations of severe disease in order to be hospitalized, the groups may no longer be appropriately matched for comparison.

**Table 4:** Comparison of IBI in both malaria and non-malaria paediatric hospital admissions

**Author**	**Malaria**	**Malaria/IBI**	**Malaria case fraction with IBI**	**Non-malarial illness**	**Non-malarial illness/IBI**	**Non-malaria case fraction with IBI**	**Malaria attributable fraction of IBI**	**Case fatality malaria**	**Case fatality malaria/IBI**
	**number**	**number**		**number**	**number**				
Akinyemi [44]	60	5	8.33%	NR	NR	-	-		
Akpede [40]	446	43	9.64%	196	24	12.24%	-2.60%	9/403 (2.2%)	3/43 (7.0%)
Bahwere [25]	182	45	24.73%	597	79	13.23%	11.49%	NR	NR
Berkley [52]	5,270	157	2.98%	6,516	677	10.39%	-7.41%	110/1,143 (9.6%)	27/83 (32.5%)
Mabey [26]	426	43	10.09%	5,240	103	1.97%	8.13%	NR	NR
Mtove [45]	947	75	7.92%	555	81	14.59%	-6.67%	NR	NR
Nadjm [5]	2,195	100	4.56%	1,444	241	16.69%	-12.13%	76/2,051 (3.7%)	13/100 (13%)
Okwara [20]	158	18	11.39%	106	14	13.21%	-1.82%	NR	NR
Sigauque [69]	10,699	621	3.15%	12,987	929	7.15%	-4.00%	NR	NR
Were [41]	506	59	11.66%	-	-	-	-	2/447 (0.4%)	2/59 (3.4%)
**Total**	20,889	1,166	5.58% (95% CI 5.34-5.50)	27,641	2,148	7.77% (95% CI 7.72-7.83)	-2.35%^a^	197/4,044 (4.9%)	45/285 (15.8%)

^a^Calculation of total case fraction excludes data from studies without non-malaria comparator group (that is, Akinyemi(43) and Were(40)). IBI, invasive bacterial infections.

### Non-severe malaria: outpatient studies

Two prospective studies (final section Table 1) describing children with non-severe malaria and concomitant IBI who did not require admission [27,28] reported vastly different case fractions of IBI (16 of 47 (34%) versus 7 of 480 (1.5%), respectively. The scarcity of this type of study in describing IBI reflects the absence of blood culture data in children in the outpatient setting.

### Other studies

Several case series, reporting prevalence and etiology of community-acquired bacteremias, have also inferred a relationship between malaria and IBI by reporting the incidence of malaria parasitemia in children with bacteremia (see Additional file 1: Table S1). In a Rwandan study, 27% of children with community-acquired bacteremia had concomitant parasitemia [29]. Malawian children with NTS bacteremia were significantly more likely to have coincident malaria and anemia than children infected with other organisms [30].

### Epidemiological studies

Three studies (see Table 2) describe and analyze data from regions in sSA where a decline in malaria burden has been paralleled by a fall in the incidence of IBI. A longitudinal case–control study in Kenya, examining host protection against bacteremia involving 1,454 cases (sickle cell trait) and 10,749 controls [31] showed that at the beginning of the study, when malaria transmission was meso-hyperendemic (mean community parasite prevalence was 29%), the bacteremia incidence rate ratio associated with malaria parasitemia was 6.69 (95% CI 1.31 to 34.3)] in children three-months- to thirteen-years-old. At this time, 62% (8.2 to 91) of bacteremia cases occurred in children with malaria infection, and sickle cell trait (HbAS) was found to be strongly protective against hospital-admission with bacteremia. Over the nine-year period, as the incidence of admission to hospital with malaria per 1,000 child-years decreased from 28.5 to 3.45, a parallel reduction in protection afforded by HbAS against bacteremia was observed. The largest reduction was in the incidence of NTS bacteremia, which was mirrored by a similar reduction in the protective effect of sickle cell trait against this organism.

In separate sites in the Gambia (Fajara and Basse) at three and four time points between 1979 and 2008, the incidence of NTS infections in children was compared to both the proportion of malaria-positive thick blood films in outpatients and the percentage of malaria admissions (Fajara only) [32]. NTS incidence declined in parallel with a reduction in the incidence of malaria. While this observation may have resulted from an overall improvement in health care in the study area, increased community use of antimicrobials or methodological bias, a similar effect was not demonstrated for invasive pneumococcal disease. Similarly, in Tanzania a decline in malaria transmission was also associated with declines in all-cause bacteremia, largely driven by an 11-fold reduction in NTS [33]. However, the Phase III muticenter RTS,S vaccine trial, involving 15,460 children six-weeks- to seventeen-months-old in seven African countries, showed that the RTS,S/AS01 achieved 35% effectiveness against severe malaria but did not show an effect on all-cause bacteremia [34].

### Mortality in malaria with concomitant IBI

In general, African children hospitalized with bacteremia have a poor prognosis [35]. The outcome of children co-infected with malaria and IBI also appears to be worse than cases of SM alone. Six studies reported mortality in children with SM with and without invasive bacteremia (Table 3). Together there were 81 fatalities, 24.1% (95% CI 18.86 to 29.36) in 336 children with malaria/IBI co-infection compared to 585 deaths in 5,760 children with malaria infection alone, 10.2% deaths (95% CI 9.33 to 10.98).

One prospective study of children admitted with clinical features of SM included 182 film-positive children: 23 had bacteremia (12.6%) and 2 of these died (8.7%) compared to 69 slide-negative children where 28 (41%) had bacteremia and of these 11 died (39.2%). Overall, morality was greatest in children younger than 18 months [22]. Other studies did not report mortality data by age group.

A retrospective study including 783 Kenyan children with SM showed a three-fold increase in mortality (10.4% to 33.3%) in those with IBI compared to those without IBI (*P* <0.001) [36]. A similar threefold increase in mortality was shown in Tanzanian children with malaria and concomitant gram-negative bacteremia [37]. The association between gram-negative organisms (especially NTS bacteremia) and mortality was reinforced by similar findings in Kenyan children [24]. In Mozambique, mortality in children with malaria and IBI co-infection increased from 4% to 22% (*P* <0.0001) [23]. Conversely, studies reported from Malawi and The Gambia found no relationship between malaria/IBI co-infection and increased mortality [38,39].

Only four of the ten studies reporting case series of febrile children with all severity malaria included data on case fatality rates for both malaria and malaria/IBI groups [5,35,40,41] (see Table 4). As with SM, case fatality was again higher in those co-infected with IBI. Overall, there were 45 deaths in 285 children with malaria/IBI (15.8%) compared to 197 deaths in 4,044 children with malaria alone (all forms) (4.9%). One study reported no association between IBI and all severity malaria [40]; however, 43% of all bacterial isolates in this study (including children with non malaria fevers) were due to *Staphyloccocus aureus*, compared to only 3% due to *Streptococcus pneumoniae* and, thus, may be not representative due to the possibility of contamination. *Were et al. *[41] reported a six-fold higher case fatality rate in malaria/IBI co-infection compared to malaria alone; however, since case fatalities were few, in total only 4 in 585 (0.7%) cases of severe malaria, this association was not significant. The study in Muheza, northeast Tanzania, describes 3,639 children admitted to a district hospital with febrile illness, where IBI increased mortality irrespective of the presence of parasitemia [5].

### Microbiological spectrum of IBI

Overall, 16 of the 22 studies in Table 1 report the prevalence and spectrum of pathogenic bacteria in children with malaria infection. Taken together for children with severe malaria five of eight studies found overall that the prevalence of Gram positive organisms exceeded that of Gram negative organisms. In three studies *S. aureus* was the major pathogen isolated [39,40,42] and in two studies >35% of isolates were *S. aureus *[22,41]. Overall, seven of fourteen hospital-based studies reporting specific microbiological data listed NTS as the most commonly cultured organism in children with malaria, with the large majority being Enteritidis and Typhimurium serovars [5,20,22,26,38,41,43]. This increased to eight studies if all EGNOs were considered [23]. *Salmonellae Typhi* was reported in isolated cases, predominantly in older children [26,44,45], with the majority of studies reporting no occurrence of *S. typhi*.

Comparing the proportion of NTS in malaria versus non-malaria infection we found that two of three SM studies with a comparator non-malaria cohort recorded a higher proportion of NTS bacteremia in the malaria group [23,24]. Of the studies reporting all malaria cases, two of three studies showed higher proportions of EGNOs in malaria compared to non-malaria infections [5,26] but NTS case numbers were only reported in one study [26]. NTS was strongly associated with recent malaria [5], which has been reported in other cohorts [46].

Among Gram-positive organisms, *S. pneumoniae* is the most common cause of IBI in children in Africa [4]. However, consensus appears to be lacking in the studies we reviewed since some studies indicate frequent co-infection with malaria [13,23,36,47] but others do not [5,22,24]. A higher prevalence of HIV and the low frequencies of sickle cell carriage in certain populations may explain these regional differences [23]. In eight studies, *S. aureus* featured as a common culture isolate in children with malaria, exceeding 35% of all isolates in five of these studies; however, there were no suggestions of a focus of infection and contamination remains a possibility.

One study has reported the case fatality of malaria complicated by Gram-positive bacteremia to be substantially lower than mortality in malaria complicated by Gram-negative bacteremia (17.2% versus 45.7%, respectively) [37]. Case fatality of the children with NTS and EGNO bacteremia appeared to be greater than other commonly isolated invasive bacterial pathogens (range 0 to 47%); however, only one study [22] made the comparison of NTS case fatality in malaria versus non-malaria groups.

### Clinical manifestations associated with malaria and concomitant IBI

A number of in-hospital studies have examined clinical manifestations associated with IBI co-morbidity, including age, SM clinical phenotype, malaria endemicity, hyperparasitemia, and underlying illness [5,22-25,38,41,43,46]. Tanzanian infants (<20 months) had higher rates of IBI co-infection (12%), particularly those <6 months old (18.5%), compared to older age groups (7.8%) [36]. A similar prevalence (15.7%) has been reported in Nigerian infants (>1- to 12-months-old) with malaria, with *E. coli* being the most common organism (36% of all organisms) [27]. IBI co-infection is also more commonly reported in areas of high malaria transmission. In an area of low transmission intensity, such as the island of Zanzibar, the incidence of concomitant IBI appears to be low (0.4%) and none had NTS [47]. The evidence surrounding hyperparasitemia as a co-factor for IBI susceptibility is inconclusive. Among children with SM and IBI in Kilifi, Kenya, 74% had hyperparasitemia (>10,000 uL) [36]. In Muheza, Tanzania, a third of children with malaria and IBI co-infection had high parasite densities [5]. In Western Kenya and Mozambique however, children with malaria and IBI (with EGNOs) were significantly more likely to have lower parasite densities [23,41].

The clinical phenotype of SM appears to be an important factor. Nigerian and Gambian children [39,43] with cerebral malaria rarely had bacterial co-infection. In Kenyan children, no association was found between the prevalence of IBI and depth of coma score [36]; whereas there was a higher case fraction with IBI in children presenting with deep breathing (a putative clinical marker of metabolic acidosis) [36]. Three studies reported that severe malarial anemia (SMA) phenotype [23,38,41] had a higher case fraction with IBI than other clinical manifestations of SM, which was also reported in studies involving all malaria [5,25,26] and this association was largely due to NTS co-infection [26,46,48]. In Malawi, compared to children with cerebral malaria (3.0%), those with SMA had a higher prevalence of IBI co-infection (11.7%), largely NTS bacteremia [38]. Of note are independent studies of NTS, conducted at the same hospital in Malawi and in Muheza, Tanzania, which described a positive association with both acute or recent malaria infection and anemia [45,48]. Similarly, in Kenyan children, three quarters of patients with NTS bacteremia complicated by anemia had evidence of a recent or current malaria infection [46].

While there is enormous epidemiological potential for co-occurrence of malaria infection, malnutrition and HIV in much of sSA, the limited data available do not support such an association. Concomitant IBI was more common in underweight children (Z-score < -2) [41] and higher parasite densities were noted in Kenyan children who were HIV positive [24]. Other studies, possibly due to limited sample size were unable to demonstrate a significant association between malaria complicated by IBI and malnutrition or HIV [37,38]. One possible explanation for a lack of association is that immunocompromised HIV-infected children may be protected from infection by the combined antimicrobial and antiparasitic properties of prophylactic co-trimoxazole [49].

Simple clinical criteria proposed by Nadjm *et al*. [5] identified 85% of malaria cases with culture-proven bacteremia. The Teule criteria included all of the following: current axillary temperature >38°C or <36°C, a positive rapid diagnostic test (RDT) or blood film for malaria, and one or more of: prostration; respiratory distress; severe anemia (hemoglobin <5 g/dL), or HIV infection.

## Discussion

Collated data examining the potential biological relationship between malaria and invasive bacterial infection across a range of studies conducted in sSA is by no means conclusive. For SM the case fraction of children with IBI co-infection was 6.4% (95% CI 5.81 to 6.98%) but with substantial between-study variability, mostly due to differences in study methodology including the case definition of SM. In prospective studies of febrile children admitted to health facilities with all-severity malaria, the case fraction with IBI was lower in children with malaria (5.6%) compared to non-malaria febrile illness (7.8%). For children with all-severity malaria (including recent infection) NTS was more commonly isolated than in non-malarial febrile illness. Added to this, several large studies [5,24] provide further support that EGNO (including NTS species) bacterial isolates are more common in children with malaria compared to non-malaria illness, but this finding was not universal. In children with SM neither those with cerebral malaria nor children with neurological complications appeared at increased risk of IBI; however, a higher proportion of children with SMA had IBI, principally NTS. Children with malaria and IBI infection had a substantially higher mortality (22%), compared to those without (12%). However, the most compelling data on the predisposition of malaria-exposed children to IBI comes from longitudinal studies capturing declining risk of IBI, spectrum of IBI organisms and risk of mortality in parallel to an epidemiological transition from high to low malaria endemicity. The largest reduction was in the incidence of NTS bacteremia, which was mirrored by a similar reduction in the protective effect of sickle cell trait against this organism. The data we have collated from hospital cohort studies corresponds with the findings reported in epidemiological studies but is less conclusive.

Teasing apart the association between malaria and IBI has been challenging for several reasons. Foremost, both malarial parasitemia and IBI are common in children presenting with fever in malaria endemic areas. Rainy seasons in the tropics predispose to blood borne infection with enteric pathogens as well as malaria outbreaks [50,51]. Hospitalized children often have overlapping etiological syndromes [52], for example, 27% of Mozambican children with radiologically confirmed pneumonias also had confirmed asexual parasitemia [30]. The limitations imposed by the lack of routine high quality microbiology services in sSA have resulted in a fragmented and patchy understanding of this association and its importance. Hard to culture pathogens, such as NTS [53], may be under-reported, conversely contamination remains a possibility in those studies reporting high incidences of staphylococcal organisms [27,39,40,42] and requires substantiating by further studies.

The findings of this review are subject to methodological limitations. Heterogeneity was high between studies. Only a few papers recorded sufficient data to allow standardization of populations. Inclusion of recent malaria infection, using antigen-based rapid diagnostic tests, has shed more light on the prevalence of co-infection and needs further investigation. We were also unable to control for differences in study design and quality of microbiological data. For many studies, the prevalence of IBI co-infecting malaria was not the primary question, making a formal meta-analysis potentially misleading. Unpublished data were not included and, therefore, our findings may have a publication bias towards studies that report high level of concordance. In children with SM the strength of a biological association with IBI is even more difficult to establish due to the paucity of data from an appropriate control population with severe life threatening illness. Our findings do, however, concur with the conclusions of the systematic review and meta-analysis of community acquired bacterial infection indicating co-infection in 6.5% of children with malaria (any type) [4].

A putative clinical association between malaria and IBI was first suggested in the 1920s in Guyana by Giglioli who observed both increased prevalence and virulence of paratyphoid fever during malaria seasons with a co-infection rate of 29% [54]. A similar rise in typhoid fever in Nigerian children during the rainy season was speculated to be linked to the increased incidence of malaria [55]. These observations of seasonal concurrence of NTS infection and malaria were further explored in Gambian children [26] showing that while stool carriage of NTS remained constant throughout the year, rises in invasive NTS infections occurred in parallel to the annual rise in malaria cases.

The propensity for co-infection with EGNOs has led to speculation that the source of the microorganisms is most likely to be from the intestine due to impaired gut barrier function [56]. A recent mouse experiment may help our understanding of the reasons why children with malaria maybe more susceptible to concomitant NTS at the biological level [57]. The production of hemoxygenase (HO-1), which is induced in response to malaria hemolysis, resulted in impaired defense by mice towards NTS infection. While not yet replicated in humans the underlying mechanisms and portal of entry of pathogens into the bloodstream include either, or a combination of, mechanical and immune-suppressive factors. Histopathological studies have demonstrated intense sequestration of *P. falciparum*-infected erythrocytes within the endothelial bed of the gut, particularly at the tip of intestinal villi [58,59]. The presence of small bowel intussusceptions in children with SM [60] has also been reported. The intense sequestration of late stage infected erythrocytes in venules and capillaries as well as the increased rigidity (or non-deformability) of non-parasitized red cells [61] both compromise microcirculatory flow to vital organs and alter endothelial cell function [62]. This may precipitate injury and impaired gut barrier function either directly or indirectly through local cytokine production, with the subsequent transfer of endotoxin and/or pathogenic bacteria into the blood stream. We have recently shown that endotoxemia is common in SM (28%) and results in temporary immune paralysis similar to that observed in patients with sepsis and experimentally-induced endotoxemia [63]. We hypothesized that the most likely origin of endotoxin was from the gut.

Although current recommendations for managing putative sepsis in children with SM are unclear [46,64], the ideal approach is not obvious [65,66]. For example, in one recent study almost 50% of bacterial isolates were resistant to the antibiotics most commonly recommended for empirical use by the WHO [5]. Moreover, in the specific case of NTS, the efficacy of gentamicin is doubtful and susceptibility testing unreliable due to the intracellular nature of this infection [67,68]. For newer antimicrobials, apart from financial considerations, there are concerns that over-use could lead to resistance. Judicious use of antibiotics may prove to be critical to those at highest risk of poor outcome.

## Conclusions

Establishing the relationship, both proof and effect, between malaria and IBI has been challenging. The accumulated evidence suggests that children with recent or acute malaria are at risk of bacterial infection, which results in an increased risk of mortality. Establishing which children with SM are at greatest risk of bacteremia would inform a policy for targeted antibiotic therapy that could substantially reduce malaria-associated mortality while minimizing the risks of excess antibiotic prescribing.

## Abbreviations

EGNO: enteric gram-negative organism; IBI: invasive bacterial infection; NTS: non-typhoidal salmonella; SM: severe malaria; SMA: severe malarial anaemia; sSA: sub Saharan Africa.

## Competing interests

The authors declare that they have no competing interests.

## Authors’ contributions

JC did the literature search, review of eligible manuscripts, constructed the first table of data and wrote the first draft of the manuscript. KM conceived the idea for a systematic review; reviewed manuscript selection; advised on design, edited tables, performed the statistical analysis and writing of the manuscript. Both authors read and approved the final manuscript.

## Authors’ information

JC is a Paediatric specialty trainee and Honorary Research Fellow at Queen Mary, University of London and was a visiting research fellow at the KEMRI-Wellcome research programme last year. KM is a Professor in Tropical Paediatric Infectious Diseases at Imperial College, London, based fulltime in East Africa at KEMRI Wellcome Trust Programme.

## Supplementary Material

Additional file 1: Table S1 Summary of excluded studies. **Table S2.** Excluded studies referring to malaria and invasive bacterial infection. Click here for file
