# Supplementary material for: Invasive bacterial co-infection in African children with Plasmodium falciparum malaria: a systematic review
Source: BMC Med. 2014 Feb 19;12:31. doi: 10.1186/1741-7015-12-31 (PMC3928319; doi:10.1186/1741-7015-12-31)
Supplement: Additional file 1: Table S1 — Summary of excluded studies. Table S2. Excluded studies referring to malaria and invasive bacterial infection. [file 1741-7015-12-31-S1.doc]

**Additional File 1: Table S1:** Summary of excluded studies

| **Total studies** | **47*** |  |
| --- | --- | --- |
| **Reason for exclusion- can be more than one** | | **No of Studies** |
| Cohort not representative of children with malaria | | 16 |
| Predominantly adults | | 14 |
| Microbiological data incomplete or not reported | | 8 |
| Unable to extract relevant data | | 7 |
| Data published elsewhere | | 6 |
| Not sub-Saharan Africa | | 4 |

**Table S2: Excluded studies referring to malaria and invasive bacterial infection**

| **Author** | **Location** | **Study Type; Primary inclusion criteria. Hospital type** | **No patients with malaria (slide +ve)** | **No of patients with IBI** | **Prevalence of concomitant IBI** | **Comments** | **Reason for Exclusion** |
| --- | --- | --- | --- | --- | --- | --- | --- |
|
| **Akpede** | Benin City, Nigeria | PCS; Fever without localizing signs.  Urban TH | 446 | 67 | 43/446 (9.6%) | Excluded: severe malnutrition, fever >7days | Data published elsewhere |
| **Akpede** | Benin City, Nigeria | PCS; Fever without localising signs.  Urban TH | 116 | 14 |  |  | Data published elsewhere |
| **Ammah** | Buea & Tiko, Cameroon | PCS; Fever with diarrhoea, headache, joint pain or nausea. Urban THs | 115 | NR | 17% had concomitant *S.typhi* and malaria | Stool cultures included | Mostly adults. Microbiology data incomplete |
| **Anderson** | Jakarta, Indonesia | PCS; Fever. Urban TH | 12 | 186 | NR |  | Not SSA. Mostly adults. Unable to extract relevant data |
| **Archibald** | Lilongwe, Malawi | PCS; Fever & prescription of anti-microbials by HCP. Urban RH | 13 | 35 | NR | During dry season. 28% HIV positive. | Unable to extract relevant data |
| **Ayoola** | Ibadan, Nigeria | PCS; Fever. Urban TH | 47 | 39 | 16/47 (34%) |  | Data published elsewhere |
| **Blomberg** | Dar es Salam, Tanzania | PCS;  Fever or IMCI danger signs. Urban TH | 354 | 255 | NR | 50% hospital acquired IBI. No data on prevalence | Unable to extract relevant data |
| **Brent** | Kilifi,  Kenya | Sub-analysis from PCS. Salmonella spp blood culture positive. Rural DH | 128 with recent malaria | 161 | 58/128 (45.3%) | NTS associated w anaemia & recent malaria (HRP2 positive, slide negative) | Cohort selected for salmonella IBI |
| **Calis** | Blantyre and Chikwawa, Malawi | Prospective case-control study; Severe anaemia. Urban referral hospital and rural district hospital | 226 | 54 | 10.1% | High prevalence of HIV | Cohort selected for all cause severe anaemia |
| **Campbell** | Bamako,  Mali | RCS; Hospitalized children. Urban TH | 427 | 98 | 98/427 (23%) | High mortality rate. Diagnosis of IBI clinical | Microbiology data incomplete |
| **Cheesborough** | Kimpese,  DR Congo | RCS; Persistent fever (5d or more) without localising signs. Rural MH | 44 | 55 | 14/44 (31.8%) | Excluded children with high parasitemia. Many prior antimalarial Rx | Cohort selected for salmonella IBI |
| **Commey** | Accra,  Ghana | RCS; Severe malaria w persistent fever (5d or more). Urban TH | 33 | 19 | 19/33 (57.6%) |  | Cohort selected for IBI |
| **Crump** | Moshi, Tanzania | PCS; Fever or history of fever in last 48hrs. Urban RH | 6 | 20 | NR | Low transmission area. Over diagnosis of malaria | Unable to extract relevant data |
| **Dougle** | Mumias, Kenya | PCS. Fever. Rural DH | 25 | 51 | 1/25 (4%) |  | Mostly adults |
| **Duggan** | Ilesa,  Nigeria | RCS; Typhoid or paratyphoid blood cx positive. Rural MH | 10 | 97 | NR  10.3% IBIs had malaria parasitaemia | Lots of pre-hospital Rx. Increase mortality associated with anaemia. | Cohort selected for salmonella IBI |
| **Dzeing-Ella** | Libreville, Gabon | PCS; Severe malaria (one or more features present). Urban RH | NR | NR | NR |  | Microbiological data absent |
| **Edwards** | Fajara, Gambia | Retrospective molecular analysis; Children with SM or UM. Three sites 40k radius of Banjul | 250 | 0 | 0 | Children with low parasite counts and suspected IBI excluded. Low transmission area. Used PCR to detect bacterial DNA. | Cohort not representative of children with malaria and IBI |
| **Endeshaw** | Gonder, Ethiopia | RCS; severe or complicated malaria. Urban TH | 104 | 15 | 14% | 78% of patients no previous exposure to malaria. High mortality | Mostly adults |
| **Enwere** | Basse and Bansang, Gambia | RCS; Outpatients with signs of ALRI or admissions w signs of IBI. Rural health centre & DH | 302 (includes HRP2  positive cases) | 295 | 197/302= 65% | Sub-analysis of pneumococcus vaccine trial. | Cohort selected for IBI |
| **Giha** | Gedarif, Sudan | PCS; Malaria.  Urban RH | 2488  110 (severe malaria) | NR | NR |  | Microbiological data absent |
| **Graham** | Blantyre, Malawi | RCS; Blood culture positive. Urban RH | 123  (82 NTS) | 403  (226 NTS) | NR  82/226 NTS (36.3%) | Recent malaria not included. Blood cx often delayed in slide positive kids. | Cohort selected for invasive NTS |
| **Green** | Zaire | RCS; suspected salmonellosis. Rural MH | NR | 206 | NR | Case fatality 66% in <6m | Cohort selected for salmonella IBI. Unable to extract relevant data |
| **Gwer** | Kilifi,  Kenya | RCS; Severe malaria. Rural DH | 1516  (3493 w features of SM) | 83 | 83/1516 = 5.5% | Strong association with mortality even if accounting for co-morbidities | Data published elsewhere |
| **Honnas** | Mumias, Kenya | PCS; Clinical symptoms suggestive of meningitis. Rural DH | 13 | NR | NR  41% patients had malaria parasitemia | Malaria did not contribute to higher mortality | Cohort selected for meningitis. Mostly adults |
| **Igharo** | Ikare Akoko, Nigeria | PCS; Fever suggestive of malaria or typhoid. Urban RH | 88 | NR | NR  18.4% malaria typhoid co-infection | Widal test used to diagnose *S.typhi* | Microbiological data absent. Mostly adults |
| **Issifou** | Lamberene & Libreville, Gabon | RCS; Hospitalised malaria. Urban RH and rural DH | 2235 | NR | NR |  | No blood cultures |
| **Lepage** | Kigali, Rwanda | PCS; Febrile presentations. Outpatient clinic | NR | 112 | NR  26.9% IBIs parasitaemic |  | Cohort selected for IBI |
| **Nesbitt** | Nairobi, Kenya | Case control study; Salmonella septicaemia. Urban TH | 10 | 60 | NR  19% NTS IBIs parasitaemia | 81% salmonella IBIs in malaria endemic areas. No typhoid patients had malaria. | Cohort selected for salmonella IBI |
| **Lesi** | Lagos, Nigeria | PCS; Cerebral malaria. Urban TH | 107 | NR | NR | IBI not recorded | Unable to extract relevant data |
| **Manning** | Madang, Papua New Guinea | RCS; Severe malaria (as defined by the WHO). Rural RH | 340 | 2 | 2/258 (0.8%) | *Candida*  and Klebsiella isolated. Not all patients had blood cultures. *P.vivax* cases included*.* | Not SSA |
| **Nsutebu** | Yaounde, Bonanjo & Tiko, Cameroon | PCS; Fever and suspected typhoid. Urban RHs | 94 | 5 | 0 |  | Mostly adults. Cohort selected for typhoid |
| **O’Dempsey** | Upper River, Gambia | PCS; Possible pneumonia, meningitis or septicaemia. Rural health clinics | 907 | 187 | NR | Children with salmonella infections had malaria pigment in leucocytes | Cohort not representative of children with malaria |
| **Oundo** | Kilifi, Kenya | RCS; All malaria admissions. Rural DH | 9147 | 67 | 67/9147 NTS (0.7%) | Only NTS reported. MDR in a large number of isolates. | Microbiology data incomplete |
| **Pattanaik** | Odisha, India | RCS; Uncomplicated and severe malaria. Urban TH | 67 | 1 | 1/67 (1.5%) |  | Not SSA. Mostly adults |
| **Peters** | Blantyre, Malawi | PCS; fever or history of fever in last 4d. Urban RH | 42 | 128 | NR |  | Mostly adults |
| **Petit** | Multisite; Kenya & Ghana | RCS; Febrile patients. Rural DHs | 176 | 73 | NR | Widal test used to diagnose *S.typhi* | Mostly adults |
| **Reddy** | Multisite; Africa | Systematic review of IBI in Africa. Sub-analysis of 9 studies reporting parasitemia | 11814 | NR | 769/11814 (6.5%)  Fungal or bacterial IBI | 2 adult & 7 paediatric studies | Data published elsewhere |
| **Schellenberg** | Ifakara, Tanzania | RCS. Malaria admissions. Rural DH | 2432 | NR | NR |  | Microbiological data absent |
| **Sigauque** | Manhica, Mozambique | PCS. X-ray confirmed pneumonia. Rural DH | 668 | 380 | 82/668 (12.3%) | 25% of severe pneumonias met definition of SM | Cohort selected for pneumonia |
| **Stein** | Harare, Zimbabwe | RCS. Malaria admissions. Urban RH | 72 | 8 | 8/72 (11.1%) | Mostly contaminants. Very few patients had blood cultures. | Mostly adults. Microbiology data incomplete |
| **Tabu** | Asembo and Nairobi, Kenya | RCS; Fever or SARI. Rural DH & urban slum clinic | NR | 385 | NR | Rural urban disparities of iNTS and S.typhi. Parallel increase of NTS and malaria in rural site. | Unable to extract relevant data |
| **Theurer** | Lilongwe, Malawi | PCS; Fever and suspected IBI. Urban TH | 44 | 19 | NR | High HIV prevalence | Mostly adults |
| **Thriemer** | Zanzibar, Tanzania | PCS. Fever. x3 Rural DHs | 28 | 79 | 1/28 (0.4%) | Region of low malaria endemicity. | Mostly adults |
| **Tripathy** | Orissa, India | PCS; Children with severe malaria. Urban TH | 374 | NR | NR | IBI not recorded | Not SSA |
| **Ukaga** | Owerri, Nigeria | Case-control study; Outpatients w uncomplicated malaria. Study site NR | 125  (42 children) | 15 | 15/42 (35.7%) | Poor quality study | Mostly adults |
| **Walsh** | Blantyre, Malawi | RCS; Febrile or very unwell children (without obvious cause). Urban RH | 4203 | 365 | NR 67/290 (23%) of IBIs had malaria parasitaemia | Excluded children with good evidence of parasitemia. | Cohort selected for IBI |

**Legend**

NR not reported

PCS Prospective Case Series

RCS Retrospective Case Series

RCT randomised controlled trial

DH district hospital

RH referral hospital

TH teaching hospital

MH mission hospital

Organisms: HIB: *Haemophylis Influenzae* NTS: non-typhoidal salmonellae; Salm spp; Salmonellae Species; SPN: *streptococcus pneumonia*; EGN enteric gram negatives; GPO gram positive organisms

**References**

1. Akpede GO, Abiodun PO, Sykes RM: **Relative contribution of bacteraemia and malaria to acute fever without localizing signs of infection in under-five children**. *Journal of Tropical Pediatrics* 1992, **38**:295-298.

2. Akpede GO, Abiodun PO, Sykes RM: **Acute fevers of unknown origin in young children in the tropics**. *Journal of Pediatrics* 1993, **122**:79-81.

3. Ammah A, Nkuo-Akenji T, Ndip R, Deas JE: **An update on concurrent malaria and typhoid fever in Cameroon**. *Transactions of the Royal Society of Tropical Medicine and Hygiene* 1999, **93**:127-129.

4. Anderson KE, Joseph SW, Nasution R, Sunoto, Butler T, Van Peenen PF, Irving GS, Saroso JS, Watten RH: **Febrile illnesses resulting in hospital admission: a bacteriological and serological study in Jakarta, Indonesia**. *American Journal of Tropical Medicine & Hygiene*, **25**:116-121.

5. Archibald LK, McDonald LC, Nwanyanwu O, Kazembe P, Dobbie H, Tokars J, Reller LB, Jarvis WR: **A hospital-based prevalence survey of bloodstream infections in febrile patients in Malawi: Implications for diagnosis and therapy**. *Journal of Infectious Diseases* 2000, **181**:1414-1420.

6. Ayoola OO, Adeyemo AA, Osinusi K: **Predictors of bacteraemia among febrile infants in Ibadan, Nigeria**. *Journal of Health, Population & Nutrition*, **20**:223-229.

7. Blomberg B, Manji KP, Urassa WK, Tamim BS, Mwakagile DS, Jureen R, Msangi V, Tellevik MG, Holberg-Petersen M, Harthug S, Maselle SY, Langeland N: **Antimicrobial resistance predicts death in Tanzanian children with bloodstream infections: a prospective cohort study**. *BMC Infectious Diseases* 2007, **7**:43.

8. Brent AJ, Oundo JO, Mwangi I, Ochola L, Lowe B, Berkley JA: **Salmonella bacteremia in Kenyan children**. *Pediatric Infectious Disease Journal* 2006, **25**:230-236.

9. Berkley JA, Bejon P, Mwangi T, Gwer S, Maitland K, Williams TN, Mohammed S, Osier F, Kinyanjui S, Fegan G, Lowe BS, English M, Peshu N, Marsh K, Newton CRJC: **HIV infection, malnutrition, and invasive bacterial infection among children with severe malaria**. *Clinical Infectious Diseases* 2009, **49**:336-343.

10. Calis JC, Phiri KS, Faragher EB, Brabin BJ, Bates I, Cuevas LE, de Haan RJ, Phiri AI, Malange P, Khoka M, Hulshof PJ, van Lieshout L, Beld MG, Teo YY, Rockett KA, Richardson A, Kwiatkowski DP, Molyneux ME, van Hensbroek MB: **Severe anemia in Malawian children**. *The New England journal of medicine* 2008, **358**:888-899.

11. Campbell JD, Sow SO, Levine MM, Kotloff KL: **The causes of hospital admission and death among children in Bamako, Mali**. *Journal of Tropical Pediatrics* 2004, **50**:158-163.

12. Cheesbrough JS, Taxman BC, Green SDR, Mewa FI, Numbi A: **Clinical definition for invasive Salmonella infection in African children**. *Pediatric Infectious Disease Journal* 1997, **16**:277-283.

13. Commey J, Quarm-Goka B, Agyepong I: **Persistent fever in severe malaria in children**. *Central African Journal of Medicine* 1994, **40**:257-260.

14. Crump JA, Ramadhani HO, Morrissey AB, Msuya LJ, Yang LY, Chow SC, Morpeth SC, Reyburn H, Njau BN, Shaw AV, Diefenthal HC, Bartlett JA, Shao JF, Schimana W, Cunningham CK, Kinabo GD: **Invasive bacterial and fungal infections among hospitalized HIV-infected and HIV-uninfected children and infants in northern Tanzania**. *Tropical Medicine and International Health* 2011, **16**:830-837.

15. Dougle M, Hendriks E, Sanders E, Dorigo-Zetsma JW: **Laboratory investigations in the diagnosis of septicaemia and malaria**. *East African Medical Journal* 1997, **74**:353-356.

16. Duggan MB, Beyer L: **Enteric fever in young Yoruba children**. *Arch Dis Child* 1975, **50**:67-71.

17. Dzeing-Ella A, Nze Obiang PC, Tchoua R, Planche T, Mboza B, Mbounja M, Muller-Roemer U, Jarvis J, Kendjo E, Ngou-Milama E, Kremsner PG, Krishna S, Kombila M: **Severe falciparum malaria in Gabonese children: clinical and laboratory features**. *Malaria journal* 2005, **4**:1.

18. Edwards MD, Morris GAJ, Burr SE, Walther M: **Evaluating the frequency of bacterial co-infections in children recruited into a malaria pathogenesis study in The Gambia, West Africa using molecular methods**. *Mol Cell Probes* 2012, **26**:151-158.

19. Endeshaw Y, Seyoum A, Amanuel B: **Clinical and laboratory features of severe and complicated falciparum malaria the experience from Gonder Hospital**. *Ethiopian Medical Journal*, **29**:21-26.

20. Enwere G, Biney E, Cheung Y, Zaman SMA, Okoko B, Oluwalana C, Vaughan A, Greenwood B, Adegbola R, Cutts FT: **Epidemiologic and clinical characteristics of community-acquired invasive bacterial infections in children aged 2-29 months in The Gambia**. *Pediatric Infectious Disease Journal* 2006, **25**:700-705.

21. Giha HA, Elghazali G, TM AE, IE AE, Eltahir EM, Baraka OZ, Khier MM, Adam I, Troye-Blomberg M, Theander TG, Elbashir MI: **Clinical pattern of severe Plasmodium falciparum malaria in Sudan in an area characterized by seasonal and unstable malaria transmission**. *Trans R Soc Trop Med Hyg* 2005, **99**:243-251.

22. Graham SM, Walsh AL, Molyneux EM, Phiri AJ, Molyneux ME: **Clinical presentation of non-typhoidal Salmonella bacteraemia in Malawian children**. *Transactions of the Royal Society of Tropical Medicine & Hygiene*, **94**:310-314.

23. Green SDR, Cheesbrough JS: **Salmonella bacteraemia among young children at a rural hospital in western Zaire**. *Annals of Tropical Paediatrics* 1993, **13**:45-53.

24. Gwer S, Newton CR, Berkley JA: **Over-diagnosis and co-morbidity of severe malaria in African children: a guide for clinicians**. *The American journal of tropical medicine and hygiene* 2007, **77**:6-13.

25. Honnas A, Petersen LT: **Bacterial meningitis in a rural Kenyan hospital**. *East African medical journal* 1998, **75**:396-401.

26. Igharo EA, Osazuwa F, Ajayi SA, Ebueku A, Igbinigie O: **Dual infection with typhoid and malaria in febrile patients in Ikare Akoko, Nigeria**. *International Journal of Tropical Medicine* 2012, **7**:49-52.

27. Issifou S, Kendjo E, Missinou MA, Matsiegui PB, Dzeing-Ella A, Dissanami FA, Kombila M, Krishna S, Kremsner PG: **Differences in presentation of severe malaria in urban and rural Gabon**. *Am J Trop Med Hyg* 2007, **77**:1015-1019.

28. Lepage P, Bogaerts J, Van Goethem C, Ntahorutaba M, Nsengumuremyi F, Hitimana DG, Vandepitte J, Butzler JP, Levy J: **Community-acquired bacteraemia in African children**. *Lancet* 1987, **1**:1458-1461.

29. Nesbitt A, Mirza NB: **Salmonella septicaemias in Kenyan children**. *Journal of Tropical Pediatrics* 1989, **35**:35-39.

30. Lesi FE, Mukhtar MY, Iroha EU, Egri-Okwaji MT: **Clinical presentation of congenital malaria at the Lagos University Teaching Hospital**. *Nigerian Journal of Clinical Practice* 2010, **13**:134-138.

31. Manning L, Laman M, Law I, Bona C, Aipit S, Teine D, Warrell J, Rosanas-Urgell A, Lin E, Kiniboro B, Vince J, Hwaiwhanje I, Karunajeewa H, Michon P, Siba P, Mueller I, Davis TME: **Features and prognosis of severe malaria caused by plasmodium falciparum, plasmodium vivax and mixed plasmodium species in Papua New Guinean children**. *PLoS ONE* 2011, **6**.

32. Nsutebu EF, Martins P, Adiogo D: **Prevalence of typhoid fever in febrile patients with symptoms clinically compatible with typhoid fever in Cameroon**. *Tropical medicine & international health : TM & IH* 2003, **8**:575-578.

33. O'Dempsey TJD, McArdle TF, Lloyd-Evans N, Baldeh I, Laurence BE, Secka O, Greenwood BM: **Importance of enteric bacteria as a cause of pneumonia, meningitis and septicemia among children in a rural community in The Gambia, West Africa**. *Pediatric Infectious Disease Journal* 1994, **13**:122-128.

34. Oundo JO, Muli F, Kariuki S, Waiyaki PG, Iijima Y, Berkley J, Kokwaro GO, Ngetsa CJ, Mwarumba S, Torto R, Lowe B: **Non-typhi salmonella in children with severe malaria**. *East Afr Med J* 2002, **79**:633-639.

35. Pattanaik SS, Tripathy R, Panda AK, Sahu AN, Das BK: **Bacteraemia in adult patients presenting with malaria in India**. *Acta tropica* 2012, **123**:136-138.

36. Peters RP, Zijlstra EE, Schijffelen MJ, Walsh AL, Joaki G, Kumwenda JJ, Kublin JG, Molyneux ME, Lewis DK: **A prospective study of bloodstream infections as cause of fever in Malawi: clinical predictors and implications for management**. *Tropical medicine & international health : TM & IH* 2004, **9**:928-934.

37. Petit PLC, Haarlem JV, Poelman M, Haverkamp MCP, Wamola IA: **Bacteraemia in patients presenting with fever**. *East African Medical Journal* 1995, **72**:116-120.

38. Reddy EA, Shaw AV, Crump JA: **Community-acquired bloodstream infections in Africa: a systematic review and meta-analysis**. *The Lancet Infectious Diseases* 2010, **10**:417-432.

39. Schellenberg D, Menendez C, Kahigwa E, Font F, Galindo C, Acosta C, Schellenberg JA, Aponte JJ, Kimario J, Urassa H, Mshinda H, Tanner M, Alonso P: **African children with malaria in an area of intense Plasmodium falciparum transmission: features on admission to the hospital and risk factors for death**. *Am J Trop Med Hyg* 1999, **61**:431-438.

40. Sigauque B, Roca A, Bassat Q, Morais L, Quinto L, Berenguera A, Machevo S, Bardaji A, Corachan M, Ribo J, Menendez C, Schuchat A, Flannery B, Soriano-Gabarro M, Alonso PL: **Severe pneumonia in Mozambican young children: Clinical and radiological characteristics and risk factors**. *Journal of Tropical Pediatrics* 2009, **55**:379-387.

41. Stein CM, Gelfand M: **The clinical features and laboratory findings in acute Plasmodium falciparum malaria in Harare, Zimbabwe**. *Central African Journal of Medicine*, **31**:166-170.

42. Tabu C, Breiman RF, Ochieng B, Aura B, Cosmas L, Audi A, Olack B, Bigogo G, Ongus JR, Fields P, Mintz E, Burton D, Oundo J, Feikin DR: **Differing burden and epidemiology of non-typhi Salmonella bacteremia in rural and urban Kenya, 2006-2009**. *PLoS ONE* 2012, **7**.

43. Theurer A, Mulenga S, Chitandale E, Neuhann F, Schutt-Gerowitt H, Fatkenheuer G: **Malaria and invasive bacterial infections as causes of fever among adult patients presenting to the medical department of a referral hospital in central Malawi**. *Tropical Medicine and International Health* 2011, **16**:59-60.

44. Thriemer K, Ley B, Ame S, von Seidlein L, de Pak G, Chang NY, Hashim R, Schmied WH, BuschClara CJL, Nixon S, Morrissey A, Puri MK, Ali M, Ochiai RL, Wierzba T, Jiddawi MS, Clemens JD, Ali SM, Deen JL: **The burden of invasive bacterial infections in Pemba, Zanzibar**. *PLoS ONE* 2012, **7**.

45. Tripathy R, Parida S, Das L, Mishra DP, Tripathy D, Das MC, Chen H, Maguire JH, Panigrahi P: **Clinical manifestations and predictors of severe malaria in Indian children**. *Pediatrics* 2007, **120**:e454-460.

46. Ukaga CN, Orji CN, Orogwu S, Nwoke BE, Anosike JC, Udujih OS, Onyeka PI, Awujo NC: **Concomitant bacteria in the blood of malaria patients in Owerri, southeastern Nigeria**. *Tanzania Health Research Bulletin* 2006, **8**:186-188.

47. Walsh AL, Phiri AJ, Graham SM, Molyneux EM, Molyneux ME: **Bacteremia in febrile Malawian children: Clinical and microbiologic features**. *Pediatric Infectious Disease Journal* 2000, **19**:312-318.
